# Supplementary figures and images for: Global antibiotic dosing strategies in hospitalised children: Characterising variation and implications for harmonisation of international guidelines
Source: PLoS One. 2021 May 27;16(5):e0252223. doi: 10.1371/journal.pone.0252223 (PMC8159011; doi:10.1371/journal.pone.0252223)

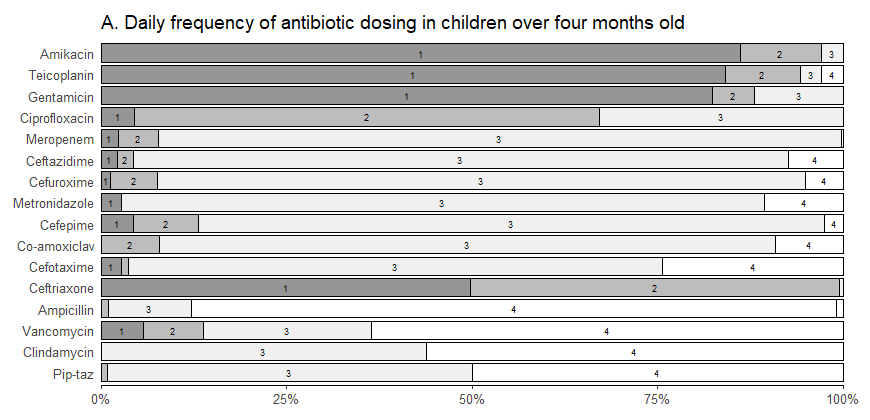

Supplement: S1 Fig — (PNG) [file pone.0252223.s001.png]

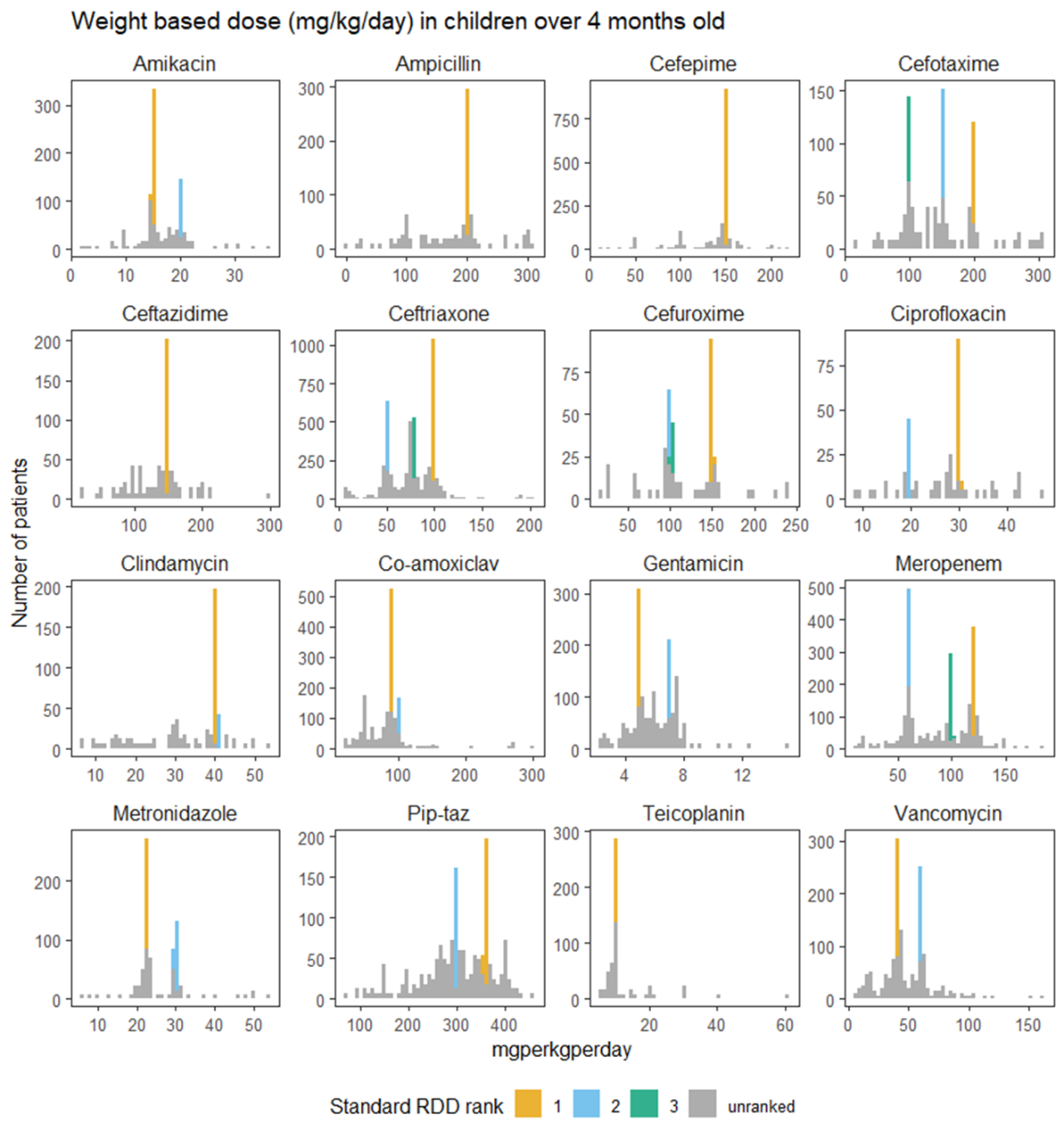

Supplement: S2 Fig — Highlighted common doses are taken from the original analyses on all children. (PNG) [file pone.0252223.s002.png]

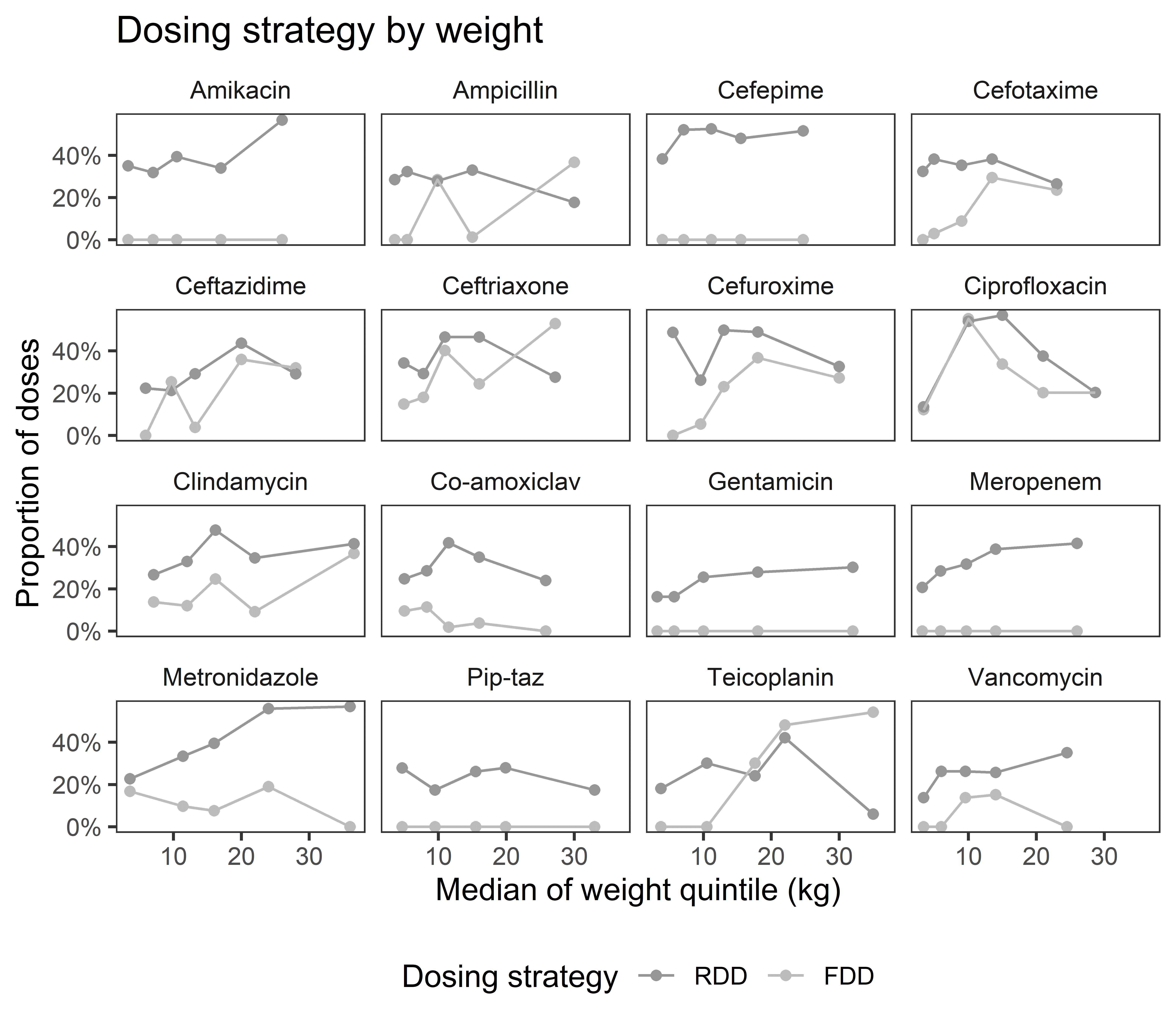

Supplement: S3 Fig — Doses consistent with both strategies are included in both categories. (PNG) [file pone.0252223.s003.png]

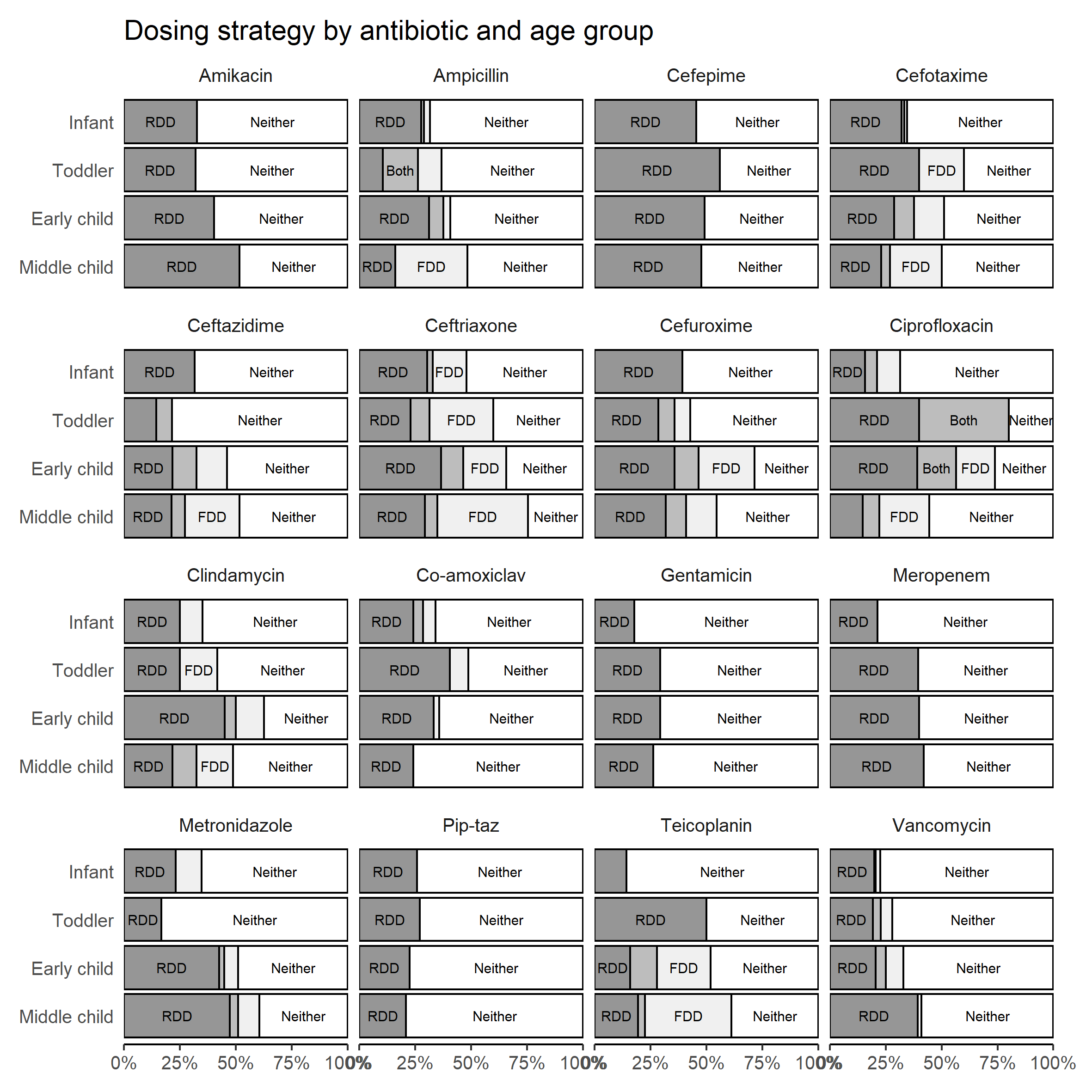

Supplement: S4 Fig — Infant: 1–12 months; Toddler 13–23 months; Early childhood: 2–5 years; Middle childhood: 6–11 years. (PNG) [file pone.0252223.s004.png]

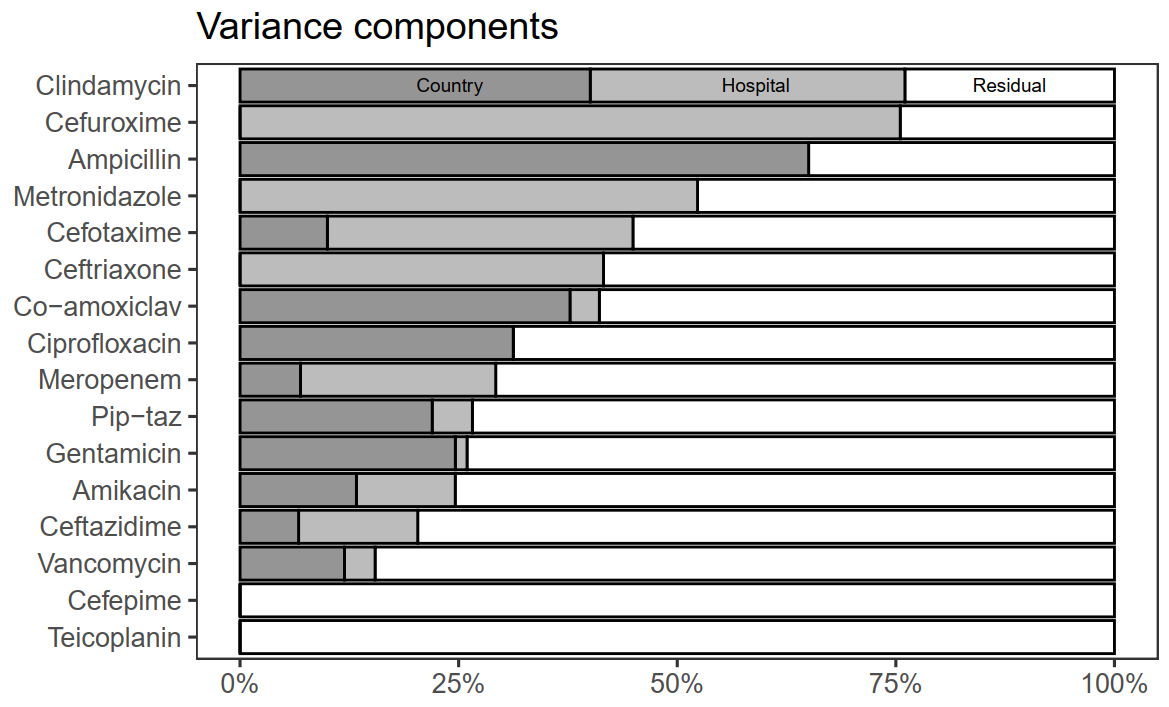

Supplement: S5 Fig — Models were run separately for each antibiotic and contained the same fixed and random effects for each model. Random effects were country and hospital within country. (PNG) [file pone.0252223.s005.png]
