# Supplementary material for: Global antibiotic dosing strategies in hospitalised children: Characterising variation and implications for harmonisation of international guidelines
Source: PLoS One. 2021 May 27;16(5):e0252223. doi: 10.1371/journal.pone.0252223 (PMC8159011; doi:10.1371/journal.pone.0252223)
Supplement: S1 Appendix — (DOCX) [file pone.0252223.s006.docx]

**Cleaning of dosing data**

Cleaning aimed to remove only obvious outliers and reduced the number of doses available for analysis from 3,469 to 3,367 (2.9%). We removed 18 doses where the unit was not recorded in mg (one dose with missing units – 2000 units of Cefotaxime - was considered to be in mg following consultation with NR); 16 doses with missing frequency or implausible frequency of 6-8 times per day. Supplementary table 1 shows the thresholds used to exclude extreme values of dose and the number of doses removed for each antibiotic. Supplementary table 2 shows the highest and lowest doses for each antibiotic once thresholds had been applied.
