# Supplementary material for: Global antibiotic dosing strategies in hospitalised children: Characterising variation and implications for harmonisation of international guidelines
Source: PLoS One. 2021 May 27;16(5):e0252223. doi: 10.1371/journal.pone.0252223 (PMC8159011; doi:10.1371/journal.pone.0252223)
Supplement: S1 Table — (DOCX) [file pone.0252223.s008.docx]

|  | **single dose max** | | **daily dose max** | | **mg/kg/day min** | | **mg/kg/day max** | |
| --- | --- | --- | --- | --- | --- | --- | --- | --- |
| **antibiotic** | **cutoff** | **N removed** | **cutoff** | **N removed** | **cutoff** | **N removed** | **cutoff** | **N removed** |
| Amikacin |  |  |  |  |  |  | 50 |  |
| Ampicillin | 8000 | 1 |  |  |  |  | 350 | 6 |
| Cefepime | 5250 | 1 | 6000 | 6 |  |  |  |  |
| Cefotaxime | 15000 | 1 |  |  |  |  | 350 | 4 |
| Ceftazidime |  |  | 12000 |  | 1 |  | 450 | 2 |
| Ceftriaxone |  |  | 6000 | 1 | 5 | 4 | 350 | 1 |
| Cefuroxime |  |  |  |  | 17.8 |  |  |  |
| Ciprofloxacin |  |  |  |  |  |  | 75 | 2 |
| Clindamycin |  |  |  |  | 5 | 1 | 150 | 9 |
| Co-amoxiclav |  |  |  |  | 17.8 | 4 |  |  |
| Gentamicin |  |  |  |  |  |  | 19 | 3 |
| Meropenem | 7200 |  |  |  | 2 | 1 | 300 | 4 |
| Metronidazole |  |  |  |  | 5 | 3 | 70 | 5 |
| Pip-taz | 6700 | 3 |  |  | 50 | 2 | 600 | 2 |
| Teicoplanin |  |  | 6000 | 1 | 2 |  |  |  |
| Vancomycin |  |  |  |  | 4 | 1 | 300 | 1 |
| **Total removed** |  | 6 |  | 8 |  | 16 |  | 39 |
