# Supplementary material for: Global antibiotic dosing strategies in hospitalised children: Characterising variation and implications for harmonisation of international guidelines
Source: PLoS One. 2021 May 27;16(5):e0252223. doi: 10.1371/journal.pone.0252223 (PMC8159011; doi:10.1371/journal.pone.0252223)
Supplement: S2 Table — (DOCX) [file pone.0252223.s009.docx]

|  | **N included** | **daily dose** | | **mg/kg/day** | |
| --- | --- | --- | --- | --- | --- |
| **antibiotic** |  | **min** | **max** | **min** | **max** |
| Amikacin | 254 | 15 | 1150 | 1.9 | 35.9 |
| Ampicillin | 158 | 10 | 8000 | 0.4 | 312 |
| Cefepime | 196 | 114 | 5400 | 12.5 | 450 |
| Cefotaxime | 170 | 80 | 9000 | 18.8 | 305.5 |
| Ceftazidime | 103 | 300 | 7500 | 20 | 300 |
| Ceftriaxone | 472 | 63 | 5200 | 6.2 | 222.2 |
| Cefuroxime | 92 | 150 | 6000 | 18.5 | 239.3 |
| Ciprofloxacin | 74 | 70 | 1200 | 8.5 | 47 |
| Clindamycin | 109 | 90 | 2256 | 6.8 | 53.5 |
| Co-amoxiclav | 263 | 99 | 5400 | 19 | 298.2 |
| Gentamicin | 215 | 4 | 400 | 2.2 | 15 |
| Meropenem | 397 | 40 | 6000 | 11.1 | 184.6 |
| Metronidazole | 132 | 19 | 1500 | 6.3 | 54.3 |
| Pip-taz | 287 | 148 | 18000 | 69.6 | 456.1 |
| Teicoplanin | 83 | 20 | 1245 | 6.1 | 61.2 |
| Vancomycin | 362 | 16 | 4000 | 6 | 160.4 |
