# Supplementary material for: Global antibiotic dosing strategies in hospitalised children: Characterising variation and implications for harmonisation of international guidelines
Source: PLoS One. 2021 May 27;16(5):e0252223. doi: 10.1371/journal.pone.0252223 (PMC8159011; doi:10.1371/journal.pone.0252223)
Supplement: S3 Table — Note that frequency once per day includes 5 doses given less than once per day (e.g. every 36h). (DOCX) [file pone.0252223.s010.docx]

|  |  | **Number of times given per day** | | | | |
| --- | --- | --- | --- | --- | --- | --- |
| **antibiotic** | **n** | **1** | **2** | **3** | **4** | **6** |
| Amikacin | 254 | 87% | 10% | 2% |  |  |
| Ampicillin | 158 |  | 3% | 11% | 85% | 1% |
| Cefepime | 196 | 4% | 12% | 83% | 2% |  |
| Cefotaxime | 170 | 2% | 6% | 64% | 29% |  |
| Ceftazidime | 103 | 2% | 2% | 89% | 7% |  |
| Ceftriaxone | 472 | 49% | 50% | 0% | 0% |  |
| Cefuroxime | 92 | 1% | 5% | 89% | 4% |  |
| Ciprofloxacin | 74 | 4% | 61% | 35% |  |  |
| Clindamycin | 109 |  |  | 45% | 55% |  |
| Co-amoxiclav | 263 |  | 10% | 80% | 10% |  |
| Gentamicin | 215 | 85% | 5% | 10% |  |  |
| Meropenem | 397 | 2% | 5% | 91% | 2% |  |
| Metronidazole | 132 | 3% | 3% | 83% | 11% |  |
| Pip-taz | 287 |  | 1% | 51% | 48% |  |
| Teicoplanin | 83 | 82% | 12% | 2% | 4% |  |
| Vancomycin | 362 | 6% | 10% | 25% | 60% |  |
