# Supplementary material for: Global antibiotic dosing strategies in hospitalised children: Characterising variation and implications for harmonisation of international guidelines
Source: PLoS One. 2021 May 27;16(5):e0252223. doi: 10.1371/journal.pone.0252223 (PMC8159011; doi:10.1371/journal.pone.0252223)
Supplement: S4 Table — Doses measured in mg/kg/day, including include a buffer of ±1% to allow for rounding before administration, and given in at least 5% of cases. (DOCX) [file pone.0252223.s011.docx]

| **antibiotic** | **n** | **1** | **2** | **3** |
| --- | --- | --- | --- | --- |
| Amikacin | 254 | 15 (30%) | 20 (10%) |  |
| Ampicillin | 158 | 200 (28%) |  |  |
| Cefepime | 196 | 150 (48%) |  |  |
| Cefotaxime | 170 | 200 (14%) | 150 (13%) | 100 (7%) |
| Ceftazidime | 103 | 150 (29%) |  |  |
| Ceftriaxone | 472 | 100 (19%) | 50 (10%) | 80 (8%) |
| Cefuroxime | 92 | 150 (22%) | 100 (10%) | 102 (10%) |
| Ciprofloxacin | 74 | 30 (24%) | 20 (12%) |  |
| Clindamycin | 109 | 40 (29%) | 41 (7%) |  |
| Co-amoxiclav | 263 | 90 (23%) | 100 (8%) |  |
| Gentamicin | 215 | 5 (15%) | 7 (8%) |  |
| Meropenem | 397 | 120 (12%) | 60 (10%) | 100 (10%) |
| Metronidazole | 132 | 22.5 (24%) | 30 (17%) |  |
| Pip-taz | 287 | 360 (13%) | 300 (10%) |  |
| Teicoplanin | 83 | 10 (24%) |  |  |
| Vancomycin | 362 | 40 (14%) | 60 (11%) |  |
