# Supplementary material for: Global antibiotic dosing strategies in hospitalised children: Characterising variation and implications for harmonisation of international guidelines
Source: PLoS One. 2021 May 27;16(5):e0252223. doi: 10.1371/journal.pone.0252223 (PMC8159011; doi:10.1371/journal.pone.0252223)
Supplement: S5 Table — Doses measured in mg/day and given in at least 5% of cases. (DOCX) [file pone.0252223.s012.docx]

| **antibiotic** | **n** | **1** | **2** | **3** |
| --- | --- | --- | --- | --- |
| Amikacin | 254 |  |  |  |
| Ampicillin | 158 | 2000 (7%) | 4000 (6%) |  |
| Cefepime | 196 |  |  |  |
| Cefotaxime | 170 | 3000 (7%) | 1200 (6%) |  |
| Ceftazidime | 103 | 3000 (8%) | 1500 (6%) | 3600 (6%) |
| Ceftriaxone | 472 | 1000 (13%) | 2000 (10%) | 500 (8%) |
| Cefuroxime | 92 | 1500 (7%) | 1800 (7%) | 4500 (5%) |
| Ciprofloxacin | 74 | 300 (11%) | 200 (9%) | 400 (8%) |
| Clindamycin | 109 | 600 (8%) | 120 (6%) | 1200 (6%) |
| Co-amoxiclav | 263 | 600 (5%) |  |  |
| Gentamicin | 215 |  |  |  |
| Meropenem | 397 |  |  |  |
| Metronidazole | 132 | 150 (5%) | 600 (5%) |  |
| Pip-taz | 287 |  |  |  |
| Teicoplanin | 83 | 400 (11%) | 200 (10%) | 160 (6%) |
| Vancomycin | 362 | 600 (6%) |  |  |
