# Supplementary material for: Global antibiotic dosing strategies in hospitalised children: Characterising variation and implications for harmonisation of international guidelines
Source: PLoS One. 2021 May 27;16(5):e0252223. doi: 10.1371/journal.pone.0252223 (PMC8159011; doi:10.1371/journal.pone.0252223)
Supplement: S6 Table — (DOCX) [file pone.0252223.s013.docx]

| **antibiotic** | **n** | **FDD** | **both** | **RDD** | **neither** |
| --- | --- | --- | --- | --- | --- |
| Amikacin | 254 | 0 (0%) | 0 (0%) | 100 (39%) | 154 (61%) |
| Ampicillin | 158 | 15 (9%) | 6 (4%) | 38 (24%) | 99 (63%) |
| Cefepime | 196 | 0 (0%) | 0 (0%) | 95 (48%) | 101 (52%) |
| Cefotaxime | 170 | 16 (9%) | 6 (4%) | 52 (31%) | 96 (56%) |
| Ceftazidime | 103 | 13 (13%) | 7 (7%) | 23 (22%) | 60 (58%) |
| Ceftriaxone | 472 | 113 (24%) | 29 (6%) | 145 (31%) | 185 (39%) |
| Cefuroxime | 92 | 11 (12%) | 6 (7%) | 32 (35%) | 43 (47%) |
| Ciprofloxacin | 74 | 12 (16%) | 9 (12%) | 18 (24%) | 35 (47%) |
| Clindamycin | 109 | 15 (14%) | 6 (6%) | 34 (31%) | 54 (50%) |
| Co-amoxiclav | 263 | 10 (4%) | 4 (2%) | 77 (29%) | 172 (65%) |
| Gentamicin | 215 | 0 (0%) | 0 (0%) | 50 (23%) | 165 (77%) |
| Meropenem | 397 | 0 (0%) | 0 (0%) | 128 (32%) | 269 (68%) |
| Metronidazole | 132 | 11 (8%) | 3 (2%) | 52 (39%) | 66 (50%) |
| Pip-taz | 287 | 0 (0%) | 0 (0%) | 67 (23%) | 220 (77%) |
| Teicoplanin | 83 | 18 (22%) | 4 (5%) | 16 (19%) | 45 (54%) |
| Vancomycin | 362 | 14 (4%) | 7 (2%) | 85 (23%) | 256 (71%) |
