# Supplementary material for: Global antibiotic dosing strategies in hospitalised children: Characterising variation and implications for harmonisation of international guidelines
Source: PLoS One. 2021 May 27;16(5):e0252223. doi: 10.1371/journal.pone.0252223 (PMC8159011; doi:10.1371/journal.pone.0252223)
Supplement: S7 Table — Models were run separately for each antibiotic and contained the same fixed and random effects for each model. Random effects were country and hospital within country. (DOCX) [file pone.0252223.s014.docx]

| **antibiotic** | **param** | **estimate** | **stderr** | **df** | **tvalue** | **pvalue** | **Upper 95%CI** | **Lower 95% CI** |
| --- | --- | --- | --- | --- | --- | --- | --- | --- |
| Amikacin | (Intercept) | 0.015 | 0.158 | 57.457 | 0.098 | 0.922 | 0.331 | -0.300 |
| Amikacin | PPS1st PPS | 0.005 | 0.081 | 158.553 | 0.067 | 0.946 | 0.166 | -0.155 |
| Amikacin | PPS2nd PPS | -0.022 | 0.068 | 168.163 | -0.323 | 0.747 | 0.113 | -0.157 |
| Amikacin | PPS3rd PPS | -0.042 | 0.064 | 165.342 | -0.655 | 0.513 | 0.085 | -0.169 |
| Amikacin | PPSPilot PPS | -0.334 | 0.184 | 82.488 | -1.814 | 0.073 | 0.032 | -0.699 |
| Amikacin | regionAfrica | -0.042 | 0.197 | 7.201 | -0.214 | 0.836 | 0.421 | -0.505 |
| Amikacin | regionAmericas | 0.050 | 0.131 | 6.757 | 0.381 | 0.715 | 0.363 | -0.263 |
| Amikacin | regionSouth-East Asia | -0.061 | 0.148 | 6.440 | -0.408 | 0.697 | 0.297 | -0.418 |
| Amikacin | regionWestern Pacific | 0.275 | 0.270 | 56.784 | 1.020 | 0.312 | 0.816 | -0.265 |
| Amikacin | sexFemale | 0.022 | 0.048 | 165.754 | 0.455 | 0.650 | 0.116 | -0.072 |
| Amikacin | age_cen | 0.097 | 0.082 | 163.885 | 1.187 | 0.237 | 0.258 | -0.064 |
| Amikacin | age_cen_sq | -0.077 | 0.058 | 163.411 | -1.330 | 0.185 | 0.037 | -0.192 |
| Amikacin | invasive_ventyes | -0.048 | 0.065 | 167.482 | -0.739 | 0.461 | 0.080 | -0.176 |
| Amikacin | comorbidityLiver | -0.103 | 0.241 | 161.622 | -0.426 | 0.671 | 0.374 | -0.580 |
| Amikacin | comorbidityOther | 0.163 | 0.094 | 166.401 | 1.730 | 0.085 | 0.349 | -0.023 |
| Amikacin | comorbidityRenal | -0.179 | 0.204 | 158.644 | -0.877 | 0.382 | 0.224 | -0.581 |
| Amikacin | other_abyes | -0.131 | 0.100 | 162.732 | -1.306 | 0.193 | 0.067 | -0.330 |
| Amikacin | other_drugsyes | 0.029 | 0.058 | 166.353 | 0.501 | 0.617 | 0.144 | -0.086 |
| Amikacin | diagnosisCentral Nervous System (CNS) infections | -1.407 | 0.327 | 157.469 | -4.304 | 0.000 | -0.761 | -2.052 |
| Amikacin | diagnosisCRBSI | -0.036 | 0.115 | 164.298 | -0.312 | 0.756 | 0.192 | -0.264 |
| Amikacin | diagnosisFebrile neutropenia/Fever | 0.039 | 0.078 | 166.327 | 0.505 | 0.614 | 0.193 | -0.114 |
| Amikacin | diagnosisGI tract infections | -0.132 | 0.123 | 161.060 | -1.074 | 0.284 | 0.111 | -0.376 |
| Amikacin | diagnosisSepsis | -0.033 | 0.068 | 163.195 | -0.488 | 0.626 | 0.101 | -0.168 |
| Amikacin | diagnosisSkin/Soft Tissue Infections (SSTI) | 0.142 | 0.153 | 166.966 | 0.929 | 0.354 | 0.443 | -0.160 |
| Amikacin | diagnosisTreatment for Surgical disease | 0.082 | 0.122 | 162.977 | 0.674 | 0.502 | 0.322 | -0.158 |
| Amikacin | diagnosisUrinary Tract Infections (UTI) | -0.142 | 0.088 | 167.535 | -1.622 | 0.107 | 0.031 | -0.316 |
| Amikacin | HAIyes | -0.044 | 0.051 | 164.345 | -0.864 | 0.389 | 0.057 | -0.145 |
| Amikacin | empiricPathogen known | 0.051 | 0.059 | 163.004 | 0.862 | 0.390 | 0.166 | -0.065 |
| Amikacin | empiricTargeted | -0.059 | 0.074 | 167.614 | -0.793 | 0.429 | 0.087 | -0.205 |
| Ampicillin | (Intercept) | 0.090 | 0.318 | 21.493 | 0.284 | 0.779 | 0.751 | -0.571 |
| Ampicillin | PPS1st PPS | 0.215 | 0.220 | 91.586 | 0.975 | 0.332 | 0.652 | -0.223 |
| Ampicillin | PPS2nd PPS | 0.061 | 0.145 | 91.415 | 0.418 | 0.677 | 0.349 | -0.228 |
| Ampicillin | PPS3rd PPS | 0.039 | 0.143 | 88.121 | 0.270 | 0.787 | 0.323 | -0.245 |
| Ampicillin | PPSPilot PPS | 0.265 | 0.339 | 86.522 | 0.782 | 0.436 | 0.938 | -0.409 |
| Ampicillin | regionAfrica | -0.696 | 0.593 | 13.378 | -1.173 | 0.261 | 0.582 | -1.973 |
| Ampicillin | regionAmericas | -0.149 | 0.423 | 12.562 | -0.352 | 0.731 | 0.768 | -1.066 |
| Ampicillin | regionSouth-East Asia | 0.203 | 0.613 | 15.239 | 0.331 | 0.745 | 1.509 | -1.103 |
| Ampicillin | regionWestern Pacific | 0.419 | 0.601 | 13.462 | 0.698 | 0.497 | 1.714 | -0.875 |
| Ampicillin | sexFemale | 0.056 | 0.111 | 89.777 | 0.503 | 0.616 | 0.276 | -0.164 |
| Ampicillin | age_cen | 0.374 | 0.193 | 89.200 | 1.940 | 0.055 | 0.758 | -0.009 |
| Ampicillin | age_cen_sq | -0.479 | 0.154 | 86.931 | -3.121 | 0.002 | -0.174 | -0.784 |
| Ampicillin | invasive_ventyes | 0.238 | 0.188 | 88.262 | 1.266 | 0.209 | 0.612 | -0.136 |
| Ampicillin | comorbidityLiver | 0.142 | 0.562 | 86.963 | 0.252 | 0.802 | 1.259 | -0.976 |
| Ampicillin | comorbidityOther | 0.013 | 0.125 | 90.551 | 0.105 | 0.916 | 0.261 | -0.235 |
| Ampicillin | other_abyes | 0.127 | 0.154 | 89.139 | 0.824 | 0.412 | 0.432 | -0.179 |
| Ampicillin | other_drugsyes | -0.110 | 0.183 | 88.001 | -0.601 | 0.550 | 0.254 | -0.474 |
| Ampicillin | diagnosisCentral Nervous System (CNS) infections | 0.132 | 0.281 | 90.528 | 0.471 | 0.639 | 0.691 | -0.426 |
| Ampicillin | diagnosisCRBSI | -0.032 | 0.425 | 94.936 | -0.076 | 0.939 | 0.811 | -0.876 |
| Ampicillin | diagnosisGI tract infections | -0.651 | 0.264 | 84.251 | -2.465 | 0.016 | -0.126 | -1.177 |
| Ampicillin | diagnosisSepsis | 0.067 | 0.203 | 89.420 | 0.328 | 0.743 | 0.470 | -0.337 |
| Ampicillin | diagnosisSkin/Soft Tissue Infections (SSTI) | 0.493 | 0.468 | 93.582 | 1.054 | 0.295 | 1.422 | -0.436 |
| Ampicillin | diagnosisTreatment for Surgical disease | -0.295 | 0.226 | 86.360 | -1.305 | 0.195 | 0.154 | -0.745 |
| Ampicillin | diagnosisUrinary Tract Infections (UTI) | -0.253 | 0.213 | 91.933 | -1.188 | 0.238 | 0.170 | -0.676 |
| Ampicillin | HAIyes | -0.258 | 0.220 | 89.733 | -1.170 | 0.245 | 0.180 | -0.696 |
| Ampicillin | empiricPathogen known | -0.006 | 0.135 | 88.980 | -0.046 | 0.963 | 0.261 | -0.274 |
| Ampicillin | empiricTargeted | 0.147 | 0.198 | 92.228 | 0.744 | 0.459 | 0.540 | -0.246 |
| Cefepime | (Intercept) | -0.131 | 0.225 | 148.000 | -0.584 | 0.560 | 0.313 | -0.575 |
| Cefepime | PPS1st PPS | 0.008 | 0.105 | 148.000 | 0.073 | 0.942 | 0.215 | -0.200 |
| Cefepime | PPS2nd PPS | 0.014 | 0.101 | 148.000 | 0.137 | 0.891 | 0.213 | -0.186 |
| Cefepime | PPS3rd PPS | 0.112 | 0.092 | 148.000 | 1.213 | 0.227 | 0.295 | -0.071 |
| Cefepime | PPSPilot PPS | 0.204 | 0.217 | 148.000 | 0.941 | 0.348 | 0.634 | -0.225 |
| Cefepime | regionAfrica | 0.282 | 0.505 | 148.000 | 0.559 | 0.577 | 1.280 | -0.715 |
| Cefepime | regionAmericas | 0.441 | 0.166 | 148.000 | 2.661 | 0.009 | 0.768 | 0.113 |
| Cefepime | regionWestern Pacific | 0.302 | 0.233 | 148.000 | 1.300 | 0.196 | 0.762 | -0.157 |
| Cefepime | sexFemale | 0.015 | 0.072 | 148.000 | 0.214 | 0.831 | 0.158 | -0.127 |
| Cefepime | age_cen | -0.033 | 0.125 | 148.000 | -0.261 | 0.794 | 0.215 | -0.280 |
| Cefepime | age_cen_sq | 0.034 | 0.099 | 148.000 | 0.349 | 0.727 | 0.229 | -0.160 |
| Cefepime | invasive_ventyes | -0.055 | 0.103 | 148.000 | -0.532 | 0.595 | 0.148 | -0.257 |
| Cefepime | comorbidityLiver | -0.471 | 0.283 | 148.000 | -1.663 | 0.098 | 0.089 | -1.030 |
| Cefepime | comorbidityOther | -0.174 | 0.151 | 148.000 | -1.152 | 0.251 | 0.124 | -0.472 |
| Cefepime | comorbidityRenal | -0.858 | 0.241 | 148.000 | -3.563 | 0.000 | -0.382 | -1.333 |
| Cefepime | other_abyes | -0.029 | 0.090 | 148.000 | -0.321 | 0.749 | 0.149 | -0.207 |
| Cefepime | other_drugsyes | -0.077 | 0.104 | 148.000 | -0.740 | 0.461 | 0.129 | -0.283 |
| Cefepime | diagnosisCentral Nervous System (CNS) infections | 0.131 | 0.177 | 148.000 | 0.740 | 0.461 | 0.480 | -0.218 |
| Cefepime | diagnosisCRBSI | 0.315 | 0.163 | 148.000 | 1.929 | 0.056 | 0.638 | -0.008 |
| Cefepime | diagnosisFebrile neutropenia/Fever | -0.006 | 0.115 | 148.000 | -0.056 | 0.956 | 0.221 | -0.234 |
| Cefepime | diagnosisGI tract infections | -0.433 | 0.343 | 148.000 | -1.264 | 0.208 | 0.244 | -1.111 |
| Cefepime | diagnosisSepsis | -0.240 | 0.100 | 148.000 | -2.394 | 0.018 | -0.042 | -0.438 |
| Cefepime | diagnosisSkin/Soft Tissue Infections (SSTI) | -0.172 | 0.163 | 148.000 | -1.055 | 0.293 | 0.150 | -0.493 |
| Cefepime | diagnosisUrinary Tract Infections (UTI) | 0.103 | 0.177 | 148.000 | 0.584 | 0.560 | 0.454 | -0.247 |
| Cefepime | HAIyes | -0.016 | 0.085 | 148.000 | -0.187 | 0.852 | 0.153 | -0.185 |
| Cefepime | empiricPathogen known | 0.019 | 0.083 | 148.000 | 0.223 | 0.824 | 0.183 | -0.146 |
| Cefepime | empiricTargeted | -0.239 | 0.147 | 148.000 | -1.629 | 0.105 | 0.051 | -0.529 |
| Cefotaxime | (Intercept) | -0.085 | 0.204 | 21.101 | -0.418 | 0.680 | 0.338 | -0.508 |
| Cefotaxime | PPS1st PPS | 0.208 | 0.308 | 95.982 | 0.676 | 0.501 | 0.819 | -0.403 |
| Cefotaxime | PPS2nd PPS | -0.013 | 0.120 | 102.402 | -0.110 | 0.913 | 0.224 | -0.251 |
| Cefotaxime | PPS3rd PPS | 0.049 | 0.109 | 98.380 | 0.448 | 0.655 | 0.264 | -0.167 |
| Cefotaxime | PPSPilot PPS | 0.359 | 0.525 | 95.460 | 0.685 | 0.495 | 1.401 | -0.682 |
| Cefotaxime | regionAfrica | 0.385 | 0.499 | 22.424 | 0.772 | 0.448 | 1.420 | -0.649 |
| Cefotaxime | regionAmericas | -0.494 | 0.337 | 24.042 | -1.466 | 0.156 | 0.201 | -1.188 |
| Cefotaxime | regionSouth-East Asia | -0.465 | 0.267 | 4.972 | -1.744 | 0.142 | 0.222 | -1.152 |
| Cefotaxime | regionWestern Pacific | 0.266 | 0.289 | 12.855 | 0.920 | 0.375 | 0.890 | -0.359 |
| Cefotaxime | sexFemale | -0.019 | 0.096 | 98.164 | -0.203 | 0.840 | 0.171 | -0.210 |
| Cefotaxime | age_cen | -0.054 | 0.170 | 101.863 | -0.319 | 0.750 | 0.283 | -0.391 |
| Cefotaxime | age_cen_sq | 0.035 | 0.136 | 99.214 | 0.260 | 0.795 | 0.304 | -0.234 |
| Cefotaxime | invasive_ventyes | -0.009 | 0.127 | 94.850 | -0.071 | 0.943 | 0.242 | -0.260 |
| Cefotaxime | comorbidityLiver | -0.136 | 0.516 | 86.835 | -0.263 | 0.793 | 0.891 | -1.162 |
| Cefotaxime | comorbidityOther | 0.044 | 0.101 | 100.695 | 0.440 | 0.661 | 0.244 | -0.155 |
| Cefotaxime | comorbidityRenal | -0.223 | 0.283 | 85.863 | -0.790 | 0.432 | 0.339 | -0.785 |
| Cefotaxime | other_abyes | -0.086 | 0.099 | 98.365 | -0.873 | 0.385 | 0.110 | -0.282 |
| Cefotaxime | other_drugsyes | -0.116 | 0.168 | 100.890 | -0.692 | 0.491 | 0.217 | -0.450 |
| Cefotaxime | diagnosisCentral Nervous System (CNS) infections | 0.549 | 0.176 | 101.573 | 3.115 | 0.002 | 0.898 | 0.199 |
| Cefotaxime | diagnosisCRBSI | 0.361 | 0.388 | 97.412 | 0.930 | 0.355 | 1.130 | -0.409 |
| Cefotaxime | diagnosisFebrile neutropenia/Fever | 0.167 | 0.526 | 98.086 | 0.318 | 0.751 | 1.210 | -0.876 |
| Cefotaxime | diagnosisGI tract infections | -0.001 | 0.262 | 103.625 | -0.003 | 0.998 | 0.518 | -0.520 |
| Cefotaxime | diagnosisSepsis | 0.071 | 0.146 | 102.952 | 0.483 | 0.630 | 0.360 | -0.219 |
| Cefotaxime | diagnosisSkin/Soft Tissue Infections (SSTI) | 0.157 | 0.269 | 94.884 | 0.581 | 0.562 | 0.692 | -0.378 |
| Cefotaxime | diagnosisTreatment for Surgical disease | 0.262 | 0.340 | 95.136 | 0.771 | 0.442 | 0.938 | -0.413 |
| Cefotaxime | diagnosisUrinary Tract Infections (UTI) | -0.361 | 0.199 | 98.294 | -1.814 | 0.073 | 0.034 | -0.756 |
| Cefotaxime | HAIyes | -0.321 | 0.137 | 100.992 | -2.345 | 0.021 | -0.049 | -0.593 |
| Cefotaxime | empiricPathogen known | 0.133 | 0.119 | 101.991 | 1.111 | 0.269 | 0.369 | -0.104 |
| Cefotaxime | empiricTargeted | 0.218 | 0.143 | 99.911 | 1.522 | 0.131 | 0.501 | -0.066 |
| Ceftazidime | (Intercept) | -0.064 | 0.237 | 28.941 | -0.268 | 0.791 | 0.422 | -0.549 |
| Ceftazidime | PPS1st PPS | 0.250 | 0.240 | 56.717 | 1.042 | 0.302 | 0.732 | -0.231 |
| Ceftazidime | PPS2nd PPS | 0.058 | 0.165 | 55.792 | 0.349 | 0.728 | 0.389 | -0.273 |
| Ceftazidime | PPS3rd PPS | 0.195 | 0.152 | 51.785 | 1.277 | 0.207 | 0.501 | -0.111 |
| Ceftazidime | PPSPilot PPS | -0.039 | 0.337 | 52.877 | -0.116 | 0.908 | 0.636 | -0.714 |
| Ceftazidime | regionAfrica | 0.535 | 0.571 | 41.528 | 0.938 | 0.354 | 1.687 | -0.617 |
| Ceftazidime | regionAmericas | -0.090 | 0.193 | 4.931 | -0.468 | 0.659 | 0.407 | -0.588 |
| Ceftazidime | regionSouth-East Asia | -0.432 | 0.274 | 3.689 | -1.574 | 0.196 | 0.356 | -1.219 |
| Ceftazidime | regionWestern Pacific | 0.290 | 0.293 | 11.726 | 0.989 | 0.342 | 0.930 | -0.350 |
| Ceftazidime | sexFemale | -0.060 | 0.110 | 53.380 | -0.542 | 0.590 | 0.161 | -0.280 |
| Ceftazidime | age_cen | -0.359 | 0.200 | 55.004 | -1.792 | 0.079 | 0.042 | -0.760 |
| Ceftazidime | age_cen_sq | 0.188 | 0.152 | 55.892 | 1.239 | 0.221 | 0.492 | -0.116 |
| Ceftazidime | invasive_ventyes | -0.108 | 0.189 | 49.622 | -0.569 | 0.572 | 0.273 | -0.488 |
| Ceftazidime | comorbidityOther | 0.243 | 0.180 | 52.778 | 1.348 | 0.183 | 0.604 | -0.118 |
| Ceftazidime | comorbidityRenal | -0.773 | 0.337 | 57.327 | -2.297 | 0.025 | -0.099 | -1.447 |
| Ceftazidime | other_abyes | 0.127 | 0.140 | 50.511 | 0.904 | 0.370 | 0.407 | -0.154 |
| Ceftazidime | other_drugsyes | -0.253 | 0.195 | 49.052 | -1.296 | 0.201 | 0.139 | -0.646 |
| Ceftazidime | diagnosisCentral Nervous System (CNS) infections | -0.030 | 0.273 | 57.347 | -0.110 | 0.913 | 0.517 | -0.577 |
| Ceftazidime | diagnosisCRBSI | -0.045 | 0.306 | 57.348 | -0.146 | 0.885 | 0.567 | -0.656 |
| Ceftazidime | diagnosisFebrile neutropenia/Fever | -0.081 | 0.246 | 56.702 | -0.328 | 0.744 | 0.412 | -0.573 |
| Ceftazidime | diagnosisGI tract infections | -0.482 | 0.236 | 54.771 | -2.040 | 0.046 | -0.008 | -0.955 |
| Ceftazidime | diagnosisSepsis | 0.015 | 0.217 | 56.557 | 0.069 | 0.946 | 0.449 | -0.419 |
| Ceftazidime | diagnosisSkin/Soft Tissue Infections (SSTI) | -0.788 | 0.301 | 52.516 | -2.618 | 0.012 | -0.184 | -1.391 |
| Ceftazidime | diagnosisTreatment for Surgical disease | 0.115 | 0.489 | 43.774 | 0.236 | 0.815 | 1.101 | -0.870 |
| Ceftazidime | diagnosisUrinary Tract Infections (UTI) | -0.148 | 0.217 | 50.137 | -0.679 | 0.500 | 0.289 | -0.584 |
| Ceftazidime | HAIyes | 0.135 | 0.165 | 50.571 | 0.822 | 0.415 | 0.466 | -0.195 |
| Ceftazidime | empiricPathogen known | -0.029 | 0.136 | 56.714 | -0.211 | 0.834 | 0.243 | -0.300 |
| Ceftazidime | empiricTargeted | -0.094 | 0.140 | 52.339 | -0.675 | 0.502 | 0.186 | -0.375 |
| Ceftriaxone | (Intercept) | -0.145 | 0.138 | 97.595 | -1.052 | 0.295 | 0.129 | -0.419 |
| Ceftriaxone | PPS1st PPS | -0.598 | 0.134 | 312.208 | -4.456 | 0.000 | -0.334 | -0.863 |
| Ceftriaxone | PPS2nd PPS | -0.227 | 0.089 | 314.946 | -2.556 | 0.011 | -0.052 | -0.402 |
| Ceftriaxone | PPS3rd PPS | 0.070 | 0.083 | 306.343 | 0.843 | 0.400 | 0.234 | -0.094 |
| Ceftriaxone | PPSPilot PPS | -0.415 | 0.256 | 323.964 | -1.618 | 0.107 | 0.090 | -0.920 |
| Ceftriaxone | regionAfrica | 0.585 | 0.305 | 32.193 | 1.922 | 0.064 | 1.206 | -0.035 |
| Ceftriaxone | regionAmericas | 0.137 | 0.224 | 36.263 | 0.614 | 0.543 | 0.591 | -0.316 |
| Ceftriaxone | regionSouth-East Asia | 0.258 | 0.213 | 36.288 | 1.212 | 0.233 | 0.690 | -0.174 |
| Ceftriaxone | regionWestern Pacific | 0.192 | 0.262 | 49.861 | 0.732 | 0.468 | 0.719 | -0.335 |
| Ceftriaxone | sexFemale | 0.070 | 0.064 | 300.162 | 1.090 | 0.277 | 0.196 | -0.056 |
| Ceftriaxone | age_cen | 0.062 | 0.124 | 311.785 | 0.497 | 0.620 | 0.305 | -0.182 |
| Ceftriaxone | age_cen_sq | -0.188 | 0.089 | 307.538 | -2.107 | 0.036 | -0.012 | -0.364 |
| Ceftriaxone | invasive_ventyes | 0.195 | 0.132 | 310.703 | 1.479 | 0.140 | 0.455 | -0.065 |
| Ceftriaxone | comorbidityOther | 0.033 | 0.075 | 317.131 | 0.434 | 0.665 | 0.181 | -0.116 |
| Ceftriaxone | comorbidityRenal | 0.072 | 0.242 | 312.959 | 0.298 | 0.766 | 0.549 | -0.404 |
| Ceftriaxone | other_abyes | 0.098 | 0.075 | 306.838 | 1.306 | 0.193 | 0.246 | -0.050 |
| Ceftriaxone | other_drugsyes | -0.051 | 0.113 | 302.027 | -0.452 | 0.652 | 0.171 | -0.273 |
| Ceftriaxone | diagnosisCentral Nervous System (CNS) infections | 0.139 | 0.102 | 307.600 | 1.356 | 0.176 | 0.340 | -0.063 |
| Ceftriaxone | diagnosisCRBSI | -0.268 | 0.421 | 286.613 | -0.636 | 0.525 | 0.560 | -1.096 |
| Ceftriaxone | diagnosisFebrile neutropenia/Fever | -0.095 | 0.202 | 320.695 | -0.468 | 0.640 | 0.303 | -0.492 |
| Ceftriaxone | diagnosisGI tract infections | -0.079 | 0.144 | 318.643 | -0.552 | 0.582 | 0.204 | -0.363 |
| Ceftriaxone | diagnosisSepsis | 0.172 | 0.096 | 309.704 | 1.792 | 0.074 | 0.360 | -0.017 |
| Ceftriaxone | diagnosisSkin/Soft Tissue Infections (SSTI) | -0.191 | 0.145 | 299.470 | -1.316 | 0.189 | 0.095 | -0.478 |
| Ceftriaxone | diagnosisTreatment for Surgical disease | -0.118 | 0.195 | 305.092 | -0.605 | 0.545 | 0.265 | -0.501 |
| Ceftriaxone | diagnosisUrinary Tract Infections (UTI) | 0.039 | 0.140 | 306.023 | 0.278 | 0.781 | 0.314 | -0.236 |
| Ceftriaxone | HAIyes | 0.088 | 0.124 | 312.354 | 0.706 | 0.480 | 0.332 | -0.156 |
| Ceftriaxone | empiricPathogen known | -0.004 | 0.083 | 320.814 | -0.046 | 0.963 | 0.159 | -0.167 |
| Ceftriaxone | empiricTargeted | 0.110 | 0.113 | 308.824 | 0.977 | 0.329 | 0.332 | -0.112 |
| Cefuroxime | (Intercept) | -0.567 | 0.284 | 30.748 | -1.995 | 0.055 | 0.013 | -1.146 |
| Cefuroxime | PPS1st PPS | 0.148 | 0.254 | 27.454 | 0.584 | 0.564 | 0.668 | -0.372 |
| Cefuroxime | PPS2nd PPS | 0.269 | 0.221 | 30.123 | 1.221 | 0.232 | 0.720 | -0.181 |
| Cefuroxime | PPS3rd PPS | 0.081 | 0.199 | 26.043 | 0.410 | 0.685 | 0.490 | -0.327 |
| Cefuroxime | regionAmericas | 0.899 | 0.456 | 16.207 | 1.972 | 0.066 | 1.864 | -0.067 |
| Cefuroxime | regionSouth-East Asia | -0.548 | 0.964 | 28.584 | -0.569 | 0.574 | 1.424 | -2.521 |
| Cefuroxime | sexFemale | -0.036 | 0.183 | 28.648 | -0.194 | 0.847 | 0.338 | -0.409 |
| Cefuroxime | age_cen | 0.127 | 0.309 | 27.189 | 0.410 | 0.685 | 0.761 | -0.508 |
| Cefuroxime | age_cen_sq | -0.332 | 0.259 | 27.947 | -1.283 | 0.210 | 0.198 | -0.862 |
| Cefuroxime | invasive_ventyes | -1.146 | 0.504 | 24.193 | -2.274 | 0.032 | -0.106 | -2.186 |
| Cefuroxime | comorbidityOther | 0.537 | 0.165 | 30.171 | 3.248 | 0.003 | 0.875 | 0.199 |
| Cefuroxime | comorbidityRenal | 0.271 | 0.847 | 19.084 | 0.320 | 0.753 | 2.044 | -1.502 |
| Cefuroxime | other_abyes | 0.428 | 0.204 | 30.112 | 2.099 | 0.044 | 0.844 | 0.012 |
| Cefuroxime | other_drugsyes | -0.405 | 0.295 | 24.142 | -1.371 | 0.183 | 0.204 | -1.013 |
| Cefuroxime | diagnosisCentral Nervous System (CNS) infections | 0.232 | 0.438 | 27.445 | 0.528 | 0.602 | 1.131 | -0.667 |
| Cefuroxime | diagnosisCRBSI | -2.114 | 0.967 | 28.737 | -2.186 | 0.037 | -0.135 | -4.093 |
| Cefuroxime | diagnosisFebrile neutropenia/Fever | 0.072 | 0.322 | 21.344 | 0.222 | 0.826 | 0.741 | -0.598 |
| Cefuroxime | diagnosisSepsis | -0.245 | 0.501 | 19.839 | -0.489 | 0.630 | 0.801 | -1.291 |
| Cefuroxime | diagnosisSkin/Soft Tissue Infections (SSTI) | -0.287 | 0.291 | 33.888 | -0.986 | 0.331 | 0.305 | -0.879 |
| Cefuroxime | diagnosisTreatment for Surgical disease | 0.541 | 0.476 | 22.928 | 1.138 | 0.267 | 1.526 | -0.443 |
| Cefuroxime | diagnosisUrinary Tract Infections (UTI) | 0.127 | 0.243 | 24.780 | 0.523 | 0.606 | 0.627 | -0.373 |
| Cefuroxime | HAIyes | 0.254 | 0.237 | 22.563 | 1.072 | 0.295 | 0.745 | -0.237 |
| Cefuroxime | empiricPathogen known | -0.055 | 0.174 | 22.880 | -0.317 | 0.754 | 0.305 | -0.416 |
| Cefuroxime | empiricTargeted | 0.334 | 0.225 | 24.584 | 1.483 | 0.151 | 0.799 | -0.130 |
| Ciprofloxacin | (Intercept) | 0.231 | 0.279 | 24.439 | 0.828 | 0.416 | 0.806 | -0.344 |
| Ciprofloxacin | PPS1st PPS | 0.027 | 0.201 | 27.374 | 0.132 | 0.896 | 0.438 | -0.385 |
| Ciprofloxacin | PPS2nd PPS | -0.111 | 0.129 | 30.270 | -0.855 | 0.399 | 0.153 | -0.375 |
| Ciprofloxacin | PPS3rd PPS | -0.121 | 0.135 | 33.737 | -0.898 | 0.376 | 0.153 | -0.396 |
| Ciprofloxacin | PPSPilot PPS | -0.577 | 0.409 | 31.273 | -1.410 | 0.168 | 0.257 | -1.412 |
| Ciprofloxacin | regionAmericas | 0.040 | 0.216 | 3.255 | 0.185 | 0.864 | 0.697 | -0.617 |
| Ciprofloxacin | regionSouth-East Asia | 0.084 | 0.257 | 3.601 | 0.326 | 0.763 | 0.831 | -0.663 |
| Ciprofloxacin | regionWestern Pacific | 0.109 | 0.576 | 24.328 | 0.190 | 0.851 | 1.297 | -1.078 |
| Ciprofloxacin | sexFemale | -0.110 | 0.103 | 33.696 | -1.077 | 0.289 | 0.098 | -0.319 |
| Ciprofloxacin | age_cen | -0.228 | 0.195 | 32.028 | -1.171 | 0.250 | 0.168 | -0.624 |
| Ciprofloxacin | age_cen_sq | -0.094 | 0.153 | 33.188 | -0.617 | 0.541 | 0.217 | -0.406 |
| Ciprofloxacin | invasive_ventyes | 0.093 | 0.123 | 29.086 | 0.756 | 0.456 | 0.345 | -0.159 |
| Ciprofloxacin | comorbidityOther | -0.016 | 0.234 | 31.542 | -0.069 | 0.945 | 0.460 | -0.492 |
| Ciprofloxacin | comorbidityRenal | -0.364 | 0.487 | 32.089 | -0.748 | 0.460 | 0.627 | -1.355 |
| Ciprofloxacin | other_abyes | 0.024 | 0.124 | 30.340 | 0.195 | 0.846 | 0.276 | -0.228 |
| Ciprofloxacin | other_drugsyes | -0.039 | 0.108 | 32.232 | -0.364 | 0.718 | 0.181 | -0.260 |
| Ciprofloxacin | diagnosisCentral Nervous System (CNS) infections | 0.179 | 0.487 | 33.000 | 0.369 | 0.715 | 1.169 | -0.811 |
| Ciprofloxacin | diagnosisCRBSI | -0.012 | 0.183 | 31.543 | -0.065 | 0.949 | 0.361 | -0.385 |
| Ciprofloxacin | diagnosisFebrile neutropenia/Fever | -0.066 | 0.198 | 30.050 | -0.333 | 0.742 | 0.339 | -0.471 |
| Ciprofloxacin | diagnosisGI tract infections | -0.140 | 0.218 | 33.519 | -0.645 | 0.523 | 0.303 | -0.584 |
| Ciprofloxacin | diagnosisSepsis | 0.257 | 0.158 | 32.962 | 1.625 | 0.114 | 0.580 | -0.065 |
| Ciprofloxacin | diagnosisSkin/Soft Tissue Infections (SSTI) | 0.437 | 0.306 | 33.373 | 1.429 | 0.162 | 1.058 | -0.185 |
| Ciprofloxacin | diagnosisTreatment for Surgical disease | -0.297 | 0.264 | 32.156 | -1.128 | 0.268 | 0.240 | -0.834 |
| Ciprofloxacin | diagnosisUrinary Tract Infections (UTI) | 0.143 | 0.315 | 30.587 | 0.453 | 0.654 | 0.785 | -0.500 |
| Ciprofloxacin | HAIyes | -0.147 | 0.134 | 32.215 | -1.099 | 0.280 | 0.125 | -0.420 |
| Ciprofloxacin | empiricPathogen known | 0.028 | 0.128 | 32.821 | 0.221 | 0.827 | 0.289 | -0.233 |
| Ciprofloxacin | empiricTargeted | -0.054 | 0.139 | 32.233 | -0.389 | 0.700 | 0.229 | -0.337 |
| Clindamycin | (Intercept) | -0.147 | 0.277 | 33.051 | -0.531 | 0.599 | 0.416 | -0.709 |
| Clindamycin | PPS1st PPS | -0.342 | 0.188 | 35.754 | -1.823 | 0.077 | 0.039 | -0.723 |
| Clindamycin | PPS2nd PPS | -0.138 | 0.118 | 41.827 | -1.173 | 0.248 | 0.099 | -0.375 |
| Clindamycin | PPS3rd PPS | 0.000 | 0.131 | 45.217 | 0.001 | 0.999 | 0.265 | -0.264 |
| Clindamycin | PPSPilot PPS | 0.063 | 0.509 | 11.325 | 0.123 | 0.904 | 1.179 | -1.053 |
| Clindamycin | regionAmericas | -0.035 | 0.326 | 12.170 | -0.108 | 0.915 | 0.673 | -0.743 |
| Clindamycin | regionSouth-East Asia | 0.159 | 0.495 | 20.002 | 0.320 | 0.752 | 1.191 | -0.874 |
| Clindamycin | regionWestern Pacific | 0.560 | 0.444 | 21.719 | 1.262 | 0.220 | 1.481 | -0.361 |
| Clindamycin | sexFemale | 0.218 | 0.109 | 44.675 | 2.012 | 0.050 | 0.437 | 0.000 |
| Clindamycin | age_cen | -0.405 | 0.212 | 47.850 | -1.907 | 0.062 | 0.022 | -0.832 |
| Clindamycin | age_cen_sq | 0.180 | 0.135 | 47.548 | 1.332 | 0.189 | 0.451 | -0.092 |
| Clindamycin | invasive_ventyes | 0.393 | 0.230 | 50.025 | 1.712 | 0.093 | 0.854 | -0.068 |
| Clindamycin | comorbidityOther | 0.187 | 0.123 | 43.821 | 1.525 | 0.134 | 0.435 | -0.060 |
| Clindamycin | comorbidityRenal | -0.224 | 0.506 | 29.800 | -0.443 | 0.661 | 0.809 | -1.258 |
| Clindamycin | other_abyes | -0.041 | 0.127 | 38.847 | -0.326 | 0.746 | 0.216 | -0.298 |
| Clindamycin | other_drugsyes | -0.520 | 0.244 | 46.854 | -2.127 | 0.039 | -0.028 | -1.011 |
| Clindamycin | diagnosisCentral Nervous System (CNS) infections | -0.229 | 0.391 | 40.395 | -0.585 | 0.562 | 0.562 | -1.019 |
| Clindamycin | diagnosisCRBSI | 0.505 | 0.388 | 37.512 | 1.304 | 0.200 | 1.290 | -0.279 |
| Clindamycin | diagnosisFebrile neutropenia/Fever | 0.832 | 0.291 | 44.332 | 2.857 | 0.006 | 1.418 | 0.245 |
| Clindamycin | diagnosisSepsis | 0.127 | 0.195 | 49.365 | 0.649 | 0.520 | 0.519 | -0.266 |
| Clindamycin | diagnosisSkin/Soft Tissue Infections (SSTI) | -0.033 | 0.157 | 46.449 | -0.208 | 0.836 | 0.284 | -0.350 |
| Clindamycin | diagnosisTreatment for Surgical disease | 0.197 | 0.180 | 40.133 | 1.097 | 0.279 | 0.561 | -0.166 |
| Clindamycin | HAIyes | 0.138 | 0.145 | 46.785 | 0.955 | 0.345 | 0.430 | -0.153 |
| Clindamycin | empiricPathogen known | -0.054 | 0.128 | 50.284 | -0.422 | 0.675 | 0.203 | -0.311 |
| Clindamycin | empiricTargeted | -0.243 | 0.174 | 46.423 | -1.392 | 0.171 | 0.108 | -0.594 |
| Co-amoxiclav | (Intercept) | 0.329 | 0.231 | 5.129 | 1.423 | 0.213 | 0.920 | -0.261 |
| Co-amoxiclav | PPS1st PPS | 0.058 | 0.218 | 156.591 | 0.266 | 0.791 | 0.489 | -0.373 |
| Co-amoxiclav | PPS2nd PPS | -0.039 | 0.108 | 168.130 | -0.361 | 0.719 | 0.175 | -0.253 |
| Co-amoxiclav | PPS3rd PPS | 0.042 | 0.105 | 164.868 | 0.398 | 0.691 | 0.250 | -0.166 |
| Co-amoxiclav | PPSPilot PPS | 1.248 | 0.331 | 156.599 | 3.770 | 0.000 | 1.902 | 0.594 |
| Co-amoxiclav | regionAfrica | 0.040 | 0.459 | 6.157 | 0.087 | 0.933 | 1.157 | -1.076 |
| Co-amoxiclav | regionAmericas | -0.487 | 0.357 | 3.764 | -1.362 | 0.249 | 0.531 | -1.504 |
| Co-amoxiclav | regionSouth-East Asia | -0.495 | 0.428 | 3.575 | -1.156 | 0.319 | 0.752 | -1.742 |
| Co-amoxiclav | regionWestern Pacific | 0.449 | 0.432 | 5.311 | 1.040 | 0.343 | 1.539 | -0.641 |
| Co-amoxiclav | sexFemale | 0.141 | 0.078 | 164.487 | 1.798 | 0.074 | 0.295 | -0.014 |
| Co-amoxiclav | age_cen | 0.036 | 0.143 | 160.470 | 0.253 | 0.801 | 0.319 | -0.247 |
| Co-amoxiclav | age_cen_sq | -0.071 | 0.117 | 158.856 | -0.604 | 0.547 | 0.161 | -0.303 |
| Co-amoxiclav | invasive_ventyes | 0.185 | 0.187 | 167.995 | 0.987 | 0.325 | 0.554 | -0.185 |
| Co-amoxiclav | comorbidityLiver | -0.209 | 0.550 | 153.878 | -0.381 | 0.704 | 0.877 | -1.296 |
| Co-amoxiclav | comorbidityOther | -0.105 | 0.099 | 169.528 | -1.059 | 0.291 | 0.090 | -0.299 |
| Co-amoxiclav | other_abyes | -0.154 | 0.122 | 164.715 | -1.263 | 0.208 | 0.087 | -0.395 |
| Co-amoxiclav | other_drugsyes | -0.332 | 0.179 | 159.438 | -1.857 | 0.065 | 0.021 | -0.685 |
| Co-amoxiclav | diagnosisCRBSI | -0.675 | 0.610 | 168.262 | -1.108 | 0.270 | 0.528 | -1.879 |
| Co-amoxiclav | diagnosisFebrile neutropenia/Fever | 0.098 | 0.347 | 138.749 | 0.283 | 0.778 | 0.785 | -0.588 |
| Co-amoxiclav | diagnosisGI tract infections | 0.012 | 0.196 | 157.996 | 0.061 | 0.951 | 0.400 | -0.376 |
| Co-amoxiclav | diagnosisSepsis | -0.039 | 0.300 | 137.898 | -0.131 | 0.896 | 0.554 | -0.633 |
| Co-amoxiclav | diagnosisSkin/Soft Tissue Infections (SSTI) | -0.446 | 0.147 | 167.040 | -3.041 | 0.003 | -0.156 | -0.735 |
| Co-amoxiclav | diagnosisTreatment for Surgical disease | -0.070 | 0.174 | 142.395 | -0.404 | 0.687 | 0.274 | -0.415 |
| Co-amoxiclav | diagnosisUrinary Tract Infections (UTI) | -0.061 | 0.187 | 158.995 | -0.327 | 0.744 | 0.309 | -0.431 |
| Co-amoxiclav | HAIyes | -0.237 | 0.163 | 164.947 | -1.457 | 0.147 | 0.084 | -0.559 |
| Co-amoxiclav | empiricPathogen known | 0.135 | 0.102 | 169.041 | 1.330 | 0.185 | 0.336 | -0.066 |
| Co-amoxiclav | empiricTargeted | 0.114 | 0.172 | 166.946 | 0.660 | 0.510 | 0.454 | -0.226 |
| Gentamicin | (Intercept) | 0.028 | 0.160 | 104.403 | 0.174 | 0.862 | 0.345 | -0.289 |
| Gentamicin | PPS1st PPS | -0.009 | 0.096 | 139.992 | -0.093 | 0.926 | 0.182 | -0.200 |
| Gentamicin | PPS2nd PPS | -0.071 | 0.055 | 146.986 | -1.286 | 0.201 | 0.038 | -0.181 |
| Gentamicin | PPS3rd PPS | -0.103 | 0.058 | 147.389 | -1.795 | 0.075 | 0.010 | -0.217 |
| Gentamicin | PPSPilot PPS | 0.040 | 0.175 | 120.557 | 0.229 | 0.820 | 0.387 | -0.307 |
| Gentamicin | regionAfrica | 0.046 | 0.143 | 11.758 | 0.325 | 0.751 | 0.358 | -0.265 |
| Gentamicin | regionAmericas | 0.128 | 0.131 | 10.675 | 0.981 | 0.348 | 0.417 | -0.160 |
| Gentamicin | regionSouth-East Asia | -0.205 | 0.202 | 21.673 | -1.013 | 0.322 | 0.215 | -0.624 |
| Gentamicin | regionWestern Pacific | -0.005 | 0.136 | 13.805 | -0.039 | 0.969 | 0.287 | -0.298 |
| Gentamicin | sexFemale | -0.017 | 0.044 | 140.487 | -0.391 | 0.696 | 0.069 | -0.104 |
| Gentamicin | age_cen | 0.199 | 0.077 | 142.393 | 2.577 | 0.011 | 0.352 | 0.046 |
| Gentamicin | age_cen_sq | -0.117 | 0.056 | 141.274 | -2.100 | 0.037 | -0.007 | -0.227 |
| Gentamicin | invasive_ventyes | 0.017 | 0.065 | 144.196 | 0.265 | 0.792 | 0.145 | -0.111 |
| Gentamicin | comorbidityLiver | 0.044 | 0.220 | 122.142 | 0.202 | 0.841 | 0.479 | -0.391 |
| Gentamicin | comorbidityOther | 0.054 | 0.062 | 149.076 | 0.877 | 0.382 | 0.176 | -0.068 |
| Gentamicin | comorbidityRenal | 0.082 | 0.302 | 148.068 | 0.273 | 0.785 | 0.680 | -0.515 |
| Gentamicin | other_abyes | 0.055 | 0.125 | 142.328 | 0.440 | 0.661 | 0.301 | -0.192 |
| Gentamicin | other_drugsyes | -0.045 | 0.064 | 135.913 | -0.707 | 0.481 | 0.081 | -0.172 |
| Gentamicin | diagnosisCentral Nervous System (CNS) infections | -0.327 | 0.179 | 146.543 | -1.828 | 0.070 | 0.026 | -0.681 |
| Gentamicin | diagnosisCRBSI | 0.071 | 0.103 | 145.147 | 0.690 | 0.491 | 0.275 | -0.132 |
| Gentamicin | diagnosisFebrile neutropenia/Fever | -0.002 | 0.098 | 133.215 | -0.024 | 0.981 | 0.192 | -0.197 |
| Gentamicin | diagnosisGI tract infections | -0.080 | 0.083 | 143.099 | -0.964 | 0.337 | 0.084 | -0.245 |
| Gentamicin | diagnosisSepsis | -0.188 | 0.069 | 150.149 | -2.716 | 0.007 | -0.051 | -0.324 |
| Gentamicin | diagnosisSkin/Soft Tissue Infections (SSTI) | -0.035 | 0.126 | 137.816 | -0.274 | 0.784 | 0.215 | -0.284 |
| Gentamicin | diagnosisTreatment for Surgical disease | -0.230 | 0.106 | 117.023 | -2.175 | 0.032 | -0.021 | -0.440 |
| Gentamicin | diagnosisUrinary Tract Infections (UTI) | 0.081 | 0.112 | 124.147 | 0.723 | 0.471 | 0.302 | -0.141 |
| Gentamicin | HAIyes | -0.142 | 0.064 | 141.792 | -2.220 | 0.028 | -0.016 | -0.269 |
| Gentamicin | empiricPathogen known | 0.055 | 0.052 | 150.149 | 1.066 | 0.288 | 0.158 | -0.047 |
| Gentamicin | empiricTargeted | 0.095 | 0.071 | 149.935 | 1.335 | 0.184 | 0.235 | -0.045 |
| Meropenem | (Intercept) | -0.177 | 0.189 | 8.655 | -0.940 | 0.373 | 0.252 | -0.607 |
| Meropenem | PPS1st PPS | -0.166 | 0.118 | 299.171 | -1.414 | 0.159 | 0.065 | -0.398 |
| Meropenem | PPS2nd PPS | -0.029 | 0.093 | 299.520 | -0.314 | 0.754 | 0.154 | -0.213 |
| Meropenem | PPS3rd PPS | -0.076 | 0.094 | 298.261 | -0.809 | 0.419 | 0.109 | -0.260 |
| Meropenem | PPSPilot PPS | 0.072 | 0.248 | 278.280 | 0.289 | 0.773 | 0.559 | -0.416 |
| Meropenem | regionAfrica | 0.531 | 0.339 | 2.535 | 1.566 | 0.231 | 1.730 | -0.669 |
| Meropenem | regionAmericas | 0.571 | 0.227 | 2.330 | 2.511 | 0.111 | 1.428 | -0.286 |
| Meropenem | regionSouth-East Asia | 0.318 | 0.250 | 2.133 | 1.275 | 0.323 | 1.331 | -0.694 |
| Meropenem | regionWestern Pacific | -0.342 | 0.348 | 15.823 | -0.981 | 0.341 | 0.397 | -1.081 |
| Meropenem | sexFemale | 0.020 | 0.068 | 296.930 | 0.296 | 0.768 | 0.154 | -0.114 |
| Meropenem | age_cen | 0.049 | 0.126 | 292.638 | 0.391 | 0.696 | 0.298 | -0.199 |
| Meropenem | age_cen_sq | -0.040 | 0.094 | 290.702 | -0.430 | 0.667 | 0.145 | -0.226 |
| Meropenem | invasive_ventyes | 0.002 | 0.077 | 299.424 | 0.021 | 0.984 | 0.153 | -0.149 |
| Meropenem | comorbidityLiver | -0.045 | 0.178 | 289.631 | -0.254 | 0.799 | 0.305 | -0.395 |
| Meropenem | comorbidityOther | -0.116 | 0.126 | 296.386 | -0.926 | 0.355 | 0.131 | -0.364 |
| Meropenem | comorbidityRenal | -0.772 | 0.220 | 296.361 | -3.503 | 0.001 | -0.338 | -1.206 |
| Meropenem | other_abyes | 0.079 | 0.078 | 294.503 | 1.010 | 0.313 | 0.231 | -0.074 |
| Meropenem | other_drugsyes | -0.007 | 0.077 | 293.172 | -0.093 | 0.926 | 0.144 | -0.158 |
| Meropenem | diagnosisCentral Nervous System (CNS) infections | 0.594 | 0.176 | 290.494 | 3.382 | 0.001 | 0.940 | 0.249 |
| Meropenem | diagnosisCRBSI | 0.437 | 0.187 | 286.870 | 2.334 | 0.020 | 0.806 | 0.068 |
| Meropenem | diagnosisFebrile neutropenia/Fever | -0.118 | 0.124 | 293.893 | -0.957 | 0.339 | 0.125 | -0.362 |
| Meropenem | diagnosisGI tract infections | -0.033 | 0.152 | 291.009 | -0.217 | 0.828 | 0.266 | -0.333 |
| Meropenem | diagnosisSepsis | 0.183 | 0.092 | 291.300 | 1.975 | 0.049 | 0.365 | 0.001 |
| Meropenem | diagnosisSkin/Soft Tissue Infections (SSTI) | -0.622 | 0.243 | 293.629 | -2.558 | 0.011 | -0.144 | -1.101 |
| Meropenem | diagnosisTreatment for Surgical disease | -0.008 | 0.188 | 297.666 | -0.045 | 0.964 | 0.362 | -0.379 |
| Meropenem | diagnosisUrinary Tract Infections (UTI) | -0.360 | 0.157 | 285.689 | -2.290 | 0.023 | -0.051 | -0.669 |
| Meropenem | HAIyes | -0.038 | 0.078 | 290.711 | -0.492 | 0.623 | 0.115 | -0.191 |
| Meropenem | empiricPathogen known | -0.026 | 0.082 | 294.305 | -0.313 | 0.755 | 0.136 | -0.187 |
| Meropenem | empiricTargeted | 0.078 | 0.090 | 296.747 | 0.858 | 0.392 | 0.255 | -0.100 |
| Metronidazole | (Intercept) | -0.429 | 0.358 | 63.239 | -1.196 | 0.236 | 0.288 | -1.145 |
| Metronidazole | PPS1st PPS | -0.049 | 0.173 | 62.101 | -0.284 | 0.778 | 0.297 | -0.395 |
| Metronidazole | PPS2nd PPS | 0.185 | 0.097 | 57.788 | 1.897 | 0.063 | 0.379 | -0.010 |
| Metronidazole | PPS3rd PPS | -0.004 | 0.092 | 56.958 | -0.044 | 0.965 | 0.180 | -0.188 |
| Metronidazole | PPSPilot PPS | -0.042 | 0.255 | 55.083 | -0.165 | 0.869 | 0.468 | -0.552 |
| Metronidazole | regionAfrica | 0.185 | 0.253 | 22.270 | 0.730 | 0.473 | 0.708 | -0.339 |
| Metronidazole | regionAmericas | 0.385 | 0.162 | 20.732 | 2.377 | 0.027 | 0.722 | 0.048 |
| Metronidazole | regionSouth-East Asia | 0.047 | 0.200 | 25.996 | 0.235 | 0.816 | 0.459 | -0.365 |
| Metronidazole | regionWestern Pacific | 0.029 | 0.340 | 19.324 | 0.084 | 0.934 | 0.739 | -0.681 |
| Metronidazole | sexFemale | 0.004 | 0.078 | 63.893 | 0.057 | 0.955 | 0.159 | -0.150 |
| Metronidazole | age_cen | 0.214 | 0.132 | 53.871 | 1.622 | 0.111 | 0.477 | -0.050 |
| Metronidazole | age_cen_sq | -0.106 | 0.084 | 50.928 | -1.259 | 0.214 | 0.063 | -0.274 |
| Metronidazole | invasive_ventyes | -0.066 | 0.118 | 54.166 | -0.560 | 0.578 | 0.171 | -0.304 |
| Metronidazole | comorbidityLiver | 0.313 | 0.429 | 44.027 | 0.729 | 0.470 | 1.177 | -0.552 |
| Metronidazole | comorbidityOther | 0.004 | 0.099 | 57.788 | 0.041 | 0.968 | 0.202 | -0.194 |
| Metronidazole | other_abyes | 0.247 | 0.206 | 54.121 | 1.197 | 0.236 | 0.661 | -0.167 |
| Metronidazole | other_drugsyes | 0.329 | 0.157 | 58.927 | 2.096 | 0.040 | 0.644 | 0.015 |
| Metronidazole | diagnosisCentral Nervous System (CNS) infections | -0.170 | 0.288 | 47.312 | -0.590 | 0.558 | 0.409 | -0.749 |
| Metronidazole | diagnosisCRBSI | 0.136 | 0.339 | 41.156 | 0.401 | 0.691 | 0.821 | -0.549 |
| Metronidazole | diagnosisFebrile neutropenia/Fever | 0.076 | 0.266 | 43.790 | 0.285 | 0.777 | 0.612 | -0.460 |
| Metronidazole | diagnosisGI tract infections | -0.059 | 0.229 | 49.734 | -0.258 | 0.797 | 0.401 | -0.519 |
| Metronidazole | diagnosisSepsis | -0.148 | 0.265 | 48.136 | -0.560 | 0.578 | 0.384 | -0.680 |
| Metronidazole | diagnosisSkin/Soft Tissue Infections (SSTI) | -0.054 | 0.283 | 57.809 | -0.191 | 0.849 | 0.512 | -0.620 |
| Metronidazole | diagnosisTreatment for Surgical disease | -0.066 | 0.242 | 49.127 | -0.272 | 0.787 | 0.420 | -0.552 |
| Metronidazole | HAIyes | 0.013 | 0.089 | 51.979 | 0.142 | 0.888 | 0.191 | -0.166 |
| Metronidazole | empiricPathogen known | 0.009 | 0.082 | 60.227 | 0.114 | 0.910 | 0.173 | -0.155 |
| Metronidazole | empiricTargeted | -0.109 | 0.126 | 57.576 | -0.865 | 0.391 | 0.144 | -0.363 |
| Pip-taz | (Intercept) | 0.081 | 0.182 | 71.506 | 0.446 | 0.657 | 0.444 | -0.282 |
| Pip-taz | PPS1st PPS | 0.082 | 0.138 | 202.402 | 0.597 | 0.551 | 0.353 | -0.189 |
| Pip-taz | PPS2nd PPS | -0.058 | 0.084 | 202.087 | -0.699 | 0.485 | 0.106 | -0.223 |
| Pip-taz | PPS3rd PPS | -0.053 | 0.080 | 194.498 | -0.663 | 0.508 | 0.105 | -0.211 |
| Pip-taz | PPSPilot PPS | 0.143 | 0.336 | 165.699 | 0.427 | 0.670 | 0.807 | -0.520 |
| Pip-taz | regionAfrica | -0.808 | 0.308 | 13.607 | -2.622 | 0.020 | -0.145 | -1.472 |
| Pip-taz | regionAmericas | 0.239 | 0.202 | 15.508 | 1.183 | 0.255 | 0.669 | -0.191 |
| Pip-taz | regionSouth-East Asia | -0.013 | 0.239 | 14.296 | -0.056 | 0.956 | 0.498 | -0.524 |
| Pip-taz | regionWestern Pacific | 0.288 | 0.272 | 17.106 | 1.057 | 0.305 | 0.861 | -0.286 |
| Pip-taz | sexFemale | -0.027 | 0.061 | 199.986 | -0.448 | 0.655 | 0.092 | -0.147 |
| Pip-taz | age_cen | 0.251 | 0.126 | 199.109 | 1.988 | 0.048 | 0.501 | 0.002 |
| Pip-taz | age_cen_sq | -0.179 | 0.082 | 196.604 | -2.198 | 0.029 | -0.018 | -0.340 |
| Pip-taz | invasive_ventyes | -0.164 | 0.091 | 195.162 | -1.799 | 0.074 | 0.016 | -0.344 |
| Pip-taz | comorbidityLiver | 0.043 | 0.226 | 200.291 | 0.190 | 0.849 | 0.489 | -0.403 |
| Pip-taz | comorbidityOther | -0.082 | 0.110 | 199.807 | -0.744 | 0.458 | 0.135 | -0.299 |
| Pip-taz | comorbidityRenal | -0.113 | 0.343 | 148.378 | -0.329 | 0.743 | 0.565 | -0.790 |
| Pip-taz | other_abyes | -0.010 | 0.071 | 175.858 | -0.141 | 0.888 | 0.130 | -0.150 |
| Pip-taz | other_drugsyes | 0.068 | 0.076 | 202.688 | 0.894 | 0.372 | 0.217 | -0.081 |
| Pip-taz | diagnosisCRBSI | -0.357 | 0.241 | 192.490 | -1.480 | 0.141 | 0.119 | -0.833 |
| Pip-taz | diagnosisFebrile neutropenia/Fever | 0.038 | 0.097 | 204.544 | 0.388 | 0.698 | 0.230 | -0.154 |
| Pip-taz | diagnosisGI tract infections | -0.030 | 0.135 | 202.480 | -0.225 | 0.822 | 0.235 | -0.296 |
| Pip-taz | diagnosisSepsis | -0.113 | 0.102 | 200.099 | -1.104 | 0.271 | 0.088 | -0.314 |
| Pip-taz | diagnosisSkin/Soft Tissue Infections (SSTI) | -0.387 | 0.170 | 200.581 | -2.276 | 0.024 | -0.052 | -0.722 |
| Pip-taz | diagnosisTreatment for Surgical disease | -0.205 | 0.167 | 203.944 | -1.226 | 0.222 | 0.125 | -0.534 |
| Pip-taz | diagnosisUrinary Tract Infections (UTI) | -0.006 | 0.162 | 203.040 | -0.038 | 0.970 | 0.314 | -0.326 |
| Pip-taz | HAIyes | -0.031 | 0.078 | 190.469 | -0.402 | 0.688 | 0.122 | -0.184 |
| Pip-taz | empiricPathogen known | 0.059 | 0.071 | 203.090 | 0.837 | 0.404 | 0.198 | -0.080 |
| Pip-taz | empiricTargeted | -0.010 | 0.118 | 201.500 | -0.086 | 0.932 | 0.222 | -0.242 |
| Teicoplanin | (Intercept) | 0.046 | 0.267 | 33.000 | 0.172 | 0.864 | 0.590 | -0.498 |
| Teicoplanin | PPS1st PPS | -0.738 | 0.573 | 33.000 | -1.288 | 0.207 | 0.428 | -1.905 |
| Teicoplanin | PPS2nd PPS | -0.163 | 0.198 | 33.000 | -0.826 | 0.415 | 0.239 | -0.565 |
| Teicoplanin | PPS3rd PPS | -0.453 | 0.197 | 33.000 | -2.295 | 0.028 | -0.051 | -0.854 |
| Teicoplanin | PPSPilot PPS | 1.088 | 0.432 | 33.000 | 2.521 | 0.017 | 1.966 | 0.210 |
| Teicoplanin | regionAmericas | 0.467 | 0.176 | 33.000 | 2.656 | 0.012 | 0.825 | 0.109 |
| Teicoplanin | regionSouth-East Asia | -0.297 | 0.445 | 33.000 | -0.668 | 0.509 | 0.608 | -1.202 |
| Teicoplanin | regionWestern Pacific | -0.003 | 0.546 | 33.000 | -0.006 | 0.996 | 1.109 | -1.115 |
| Teicoplanin | sexFemale | 0.091 | 0.117 | 33.000 | 0.774 | 0.444 | 0.330 | -0.148 |
| Teicoplanin | age_cen | -0.279 | 0.231 | 33.000 | -1.211 | 0.235 | 0.190 | -0.748 |
| Teicoplanin | age_cen_sq | 0.277 | 0.160 | 33.000 | 1.733 | 0.092 | 0.602 | -0.048 |
| Teicoplanin | invasive_ventyes | 0.273 | 0.182 | 33.000 | 1.500 | 0.143 | 0.642 | -0.097 |
| Teicoplanin | comorbidityOther | 0.244 | 0.207 | 33.000 | 1.179 | 0.247 | 0.664 | -0.177 |
| Teicoplanin | comorbidityRenal | 0.232 | 0.428 | 33.000 | 0.542 | 0.592 | 1.103 | -0.639 |
| Teicoplanin | other_abyes | 0.103 | 0.196 | 33.000 | 0.524 | 0.604 | 0.501 | -0.296 |
| Teicoplanin | other_drugsyes | -0.218 | 0.149 | 33.000 | -1.459 | 0.154 | 0.086 | -0.522 |
| Teicoplanin | diagnosisCentral Nervous System (CNS) infections | -0.022 | 0.368 | 33.000 | -0.060 | 0.953 | 0.727 | -0.771 |
| Teicoplanin | diagnosisCRBSI | -0.326 | 0.231 | 33.000 | -1.411 | 0.167 | 0.144 | -0.796 |
| Teicoplanin | diagnosisFebrile neutropenia/Fever | -0.207 | 0.220 | 33.000 | -0.940 | 0.354 | 0.240 | -0.654 |
| Teicoplanin | diagnosisGI tract infections | -0.005 | 0.296 | 33.000 | -0.016 | 0.987 | 0.597 | -0.607 |
| Teicoplanin | diagnosisSepsis | -0.069 | 0.226 | 33.000 | -0.304 | 0.763 | 0.391 | -0.528 |
| Teicoplanin | diagnosisSkin/Soft Tissue Infections (SSTI) | -0.444 | 0.349 | 33.000 | -1.274 | 0.212 | 0.265 | -1.154 |
| Teicoplanin | diagnosisTreatment for Surgical disease | -0.993 | 0.395 | 33.000 | -2.515 | 0.017 | -0.190 | -1.796 |
| Teicoplanin | HAIyes | -0.266 | 0.159 | 33.000 | -1.669 | 0.105 | 0.058 | -0.590 |
| Teicoplanin | empiricPathogen known | 0.121 | 0.148 | 33.000 | 0.816 | 0.420 | 0.423 | -0.181 |
| Teicoplanin | empiricTargeted | -0.089 | 0.169 | 33.000 | -0.528 | 0.601 | 0.254 | -0.432 |
| Vancomycin | (Intercept) | 0.007 | 0.230 | 105.191 | 0.029 | 0.977 | 0.462 | -0.449 |
| Vancomycin | PPS1st PPS | 0.008 | 0.140 | 222.124 | 0.055 | 0.956 | 0.283 | -0.268 |
| Vancomycin | PPS2nd PPS | -0.033 | 0.097 | 262.250 | -0.337 | 0.737 | 0.158 | -0.223 |
| Vancomycin | PPS3rd PPS | 0.096 | 0.098 | 257.578 | 0.978 | 0.329 | 0.288 | -0.097 |
| Vancomycin | PPSPilot PPS | 0.277 | 0.255 | 109.698 | 1.085 | 0.280 | 0.782 | -0.229 |
| Vancomycin | regionAfrica | 0.744 | 0.338 | 12.278 | 2.201 | 0.048 | 1.479 | 0.009 |
| Vancomycin | regionAmericas | 0.217 | 0.186 | 7.551 | 1.166 | 0.279 | 0.651 | -0.217 |
| Vancomycin | regionSouth-East Asia | 0.606 | 0.240 | 9.322 | 2.527 | 0.032 | 1.145 | 0.066 |
| Vancomycin | regionWestern Pacific | 0.651 | 0.230 | 12.137 | 2.833 | 0.015 | 1.151 | 0.151 |
| Vancomycin | sexFemale | -0.039 | 0.072 | 257.713 | -0.550 | 0.583 | 0.102 | -0.180 |
| Vancomycin | age_cen | 0.318 | 0.134 | 257.573 | 2.371 | 0.018 | 0.582 | 0.054 |
| Vancomycin | age_cen_sq | -0.230 | 0.108 | 261.134 | -2.120 | 0.035 | -0.016 | -0.443 |
| Vancomycin | invasive_ventyes | -0.028 | 0.080 | 262.184 | -0.352 | 0.725 | 0.129 | -0.185 |
| Vancomycin | comorbidityLiver | -0.169 | 0.230 | 258.967 | -0.734 | 0.464 | 0.284 | -0.622 |
| Vancomycin | comorbidityOther | -0.072 | 0.118 | 259.130 | -0.610 | 0.542 | 0.160 | -0.304 |
| Vancomycin | comorbidityRenal | -0.919 | 0.244 | 257.211 | -3.766 | 0.000 | -0.438 | -1.399 |
| Vancomycin | other_abyes | -0.015 | 0.133 | 260.581 | -0.111 | 0.912 | 0.247 | -0.277 |
| Vancomycin | other_drugsyes | -0.110 | 0.082 | 263.601 | -1.348 | 0.179 | 0.051 | -0.271 |
| Vancomycin | diagnosisCentral Nervous System (CNS) infections | 0.297 | 0.137 | 257.609 | 2.169 | 0.031 | 0.567 | 0.027 |
| Vancomycin | diagnosisCRBSI | -0.095 | 0.135 | 264.822 | -0.701 | 0.484 | 0.171 | -0.360 |
| Vancomycin | diagnosisFebrile neutropenia/Fever | -0.018 | 0.161 | 262.043 | -0.111 | 0.911 | 0.299 | -0.335 |
| Vancomycin | diagnosisGI tract infections | -0.260 | 0.213 | 263.063 | -1.225 | 0.222 | 0.158 | -0.679 |
| Vancomycin | diagnosisSepsis | -0.223 | 0.119 | 264.913 | -1.869 | 0.063 | 0.012 | -0.458 |
| Vancomycin | diagnosisSkin/Soft Tissue Infections (SSTI) | -0.083 | 0.151 | 262.835 | -0.550 | 0.583 | 0.214 | -0.380 |
| Vancomycin | diagnosisTreatment for Surgical disease | -0.093 | 0.240 | 263.760 | -0.386 | 0.700 | 0.381 | -0.566 |
| Vancomycin | diagnosisUrinary Tract Infections (UTI) | -0.595 | 0.367 | 264.478 | -1.620 | 0.106 | 0.128 | -1.319 |
| Vancomycin | HAIyes | -0.182 | 0.087 | 259.925 | -2.079 | 0.039 | -0.010 | -0.354 |
| Vancomycin | empiricPathogen known | 0.117 | 0.092 | 234.498 | 1.271 | 0.205 | 0.299 | -0.065 |
| Vancomycin | empiricTargeted | 0.418 | 0.107 | 261.619 | 3.909 | 0.000 | 0.629 | 0.208 |
