# Supplementary material for: Global antibiotic dosing strategies in hospitalised children: Characterising variation and implications for harmonisation of international guidelines
Source: PLoS One. 2021 May 27;16(5):e0252223. doi: 10.1371/journal.pone.0252223 (PMC8159011; doi:10.1371/journal.pone.0252223)
Supplement: S8 Table — Models were run separately for each antibiotic and contained the same fixed and random effects for each model. Random effects were country and hospital within country. (DOCX) [file pone.0252223.s015.docx]

| **antibiotic** | **factor** | **var** | **percent** |
| --- | --- | --- | --- |
| Amikacin | hospital | 0.013 | 11% |
| Amikacin | country | 0.015 | 13% |
| Amikacin | Residual | 0.086 | 75% |
| Ampicillin | hospital | 0 | 0% |
| Ampicillin | country | 0.436 | 65% |
| Ampicillin | Residual | 0.234 | 35% |
| Cefepime | hospital | 0 | 0% |
| Cefepime | country | 0 | 0% |
| Cefepime | Residual | 0.185 | 100% |
| Cefotaxime | hospital | 0.114 | 35% |
| Cefotaxime | country | 0.033 | 10% |
| Cefotaxime | Residual | 0.18 | 55% |
| Ceftazidime | hospital | 0.029 | 14% |
| Ceftazidime | country | 0.014 | 7% |
| Ceftazidime | Residual | 0.168 | 80% |
| Ceftriaxone | hospital | 0.202 | 42% |
| Ceftriaxone | country | 0 | 0% |
| Ceftriaxone | Residual | 0.284 | 58% |
| Cefuroxime | hospital | 0.454 | 76% |
| Cefuroxime | country | 0 | 0% |
| Cefuroxime | Residual | 0.147 | 24% |
| Ciprofloxacin | hospital | 0 | 0% |
| Ciprofloxacin | country | 0.041 | 31% |
| Ciprofloxacin | Residual | 0.091 | 69% |
| Clindamycin | hospital | 0.136 | 36% |
| Clindamycin | country | 0.152 | 40% |
| Clindamycin | Residual | 0.091 | 24% |
| Co-amoxiclav | hospital | 0.015 | 3% |
| Co-amoxiclav | country | 0.163 | 38% |
| Co-amoxiclav | Residual | 0.255 | 59% |
| Gentamicin | hospital | 0.001 | 1% |
| Gentamicin | country | 0.023 | 25% |
| Gentamicin | Residual | 0.068 | 74% |
| Meropenem | hospital | 0.101 | 22% |
| Meropenem | country | 0.031 | 7% |
| Meropenem | Residual | 0.319 | 71% |
| Metronidazole | hospital | 0.069 | 52% |
| Metronidazole | country | 0 | 0% |
| Metronidazole | Residual | 0.063 | 48% |
| Pip-taz | hospital | 0.011 | 5% |
| Pip-taz | country | 0.055 | 22% |
| Pip-taz | Residual | 0.183 | 73% |
| Teicoplanin | hospital | 0 | 0% |
| Teicoplanin | country | 0 | 0% |
| Teicoplanin | Residual | 0.13 | 100% |
| Vancomycin | hospital | 0.014 | 4% |
| Vancomycin | country | 0.047 | 12% |
| Vancomycin | Residual | 0.332 | 85% |
